# Merkel Cell Polyomavirus Small T Antigen Drives Cell Motility via Rho-GTPase-Induced Filopodium Formation

**DOI:** 10.1128/JVI.00940-17

**Published:** 2018-01-02

**Authors:** Gabrielė Stakaitytė, Nnenna Nwogu, Samuel J. Dobson, Laura M. Knight, Christopher W. Wasson, Francisco J. Salguero, David J. Blackbourn, G. Eric Blair, Jamel Mankouri, Andrew Macdonald, Adrian Whitehouse

**Affiliations:** a School of Molecular and Cellular Biology, Faculty of Biological Sciences, University of Leeds, Leeds, United Kingdom; b Astbury Centre for Structural Molecular Biology, University of Leeds, Leeds, United Kingdom; c School of Veterinary Medicine, University of Surrey, Surrey, United Kingdom; d School of Biosciences and Medicine, University of Surrey, Surrey, United Kingdom; International Centre for Genetic Engineering and Biotechnology

**Keywords:** DNA viruses, tumor virus, cell motility, cell migration, Merkel cell, polyomavirus

## Abstract

Cell motility and migration is a complex, multistep, and multicomponent process intrinsic to progression and metastasis. Motility is dependent on the activities of integrin receptors and Rho family GTPases, resulting in the remodeling of the actin cytoskeleton and formation of various motile actin-based protrusions. Merkel cell carcinoma (MCC) is an aggressive skin cancer with a high likelihood of recurrence and metastasis. Merkel cell polyomavirus (MCPyV) is associated with the majority of MCC cases, and MCPyV-induced tumorigenesis largely depends on the expression of the small tumor antigen (ST). Since the discovery of MCPyV, a number of mechanisms have been suggested to account for replication and tumorigenesis, but to date, little is known about potential links between MCPyV T antigen expression and the metastatic nature of MCC. Previously, we described the action of MCPyV ST on the microtubule network and how it impacts cell motility and migration. Here, we demonstrate that MCPyV ST affects the actin cytoskeleton to promote the formation of filopodia through a mechanism involving the catalytic subunit of protein phosphatase 4 (PP4C). We also show that MCPyV ST-induced cell motility is dependent upon the activities of the Rho family GTPases Cdc42 and RhoA. In addition, our results indicate that the MCPyV ST-PP4C interaction results in the dephosphorylation of β_1_ integrin, likely driving the cell motility pathway. These findings describe a novel mechanism by which a tumor virus induces cell motility, which may ultimately lead to cancer metastasis, and provides opportunities and strategies for targeted interventions for disseminated MCC.

**IMPORTANCE** Merkel cell polyomavirus (MCPyV) is the most recently discovered human tumor virus. It causes the majority of cases of Merkel cell carcinoma (MCC), an aggressive skin cancer. However, the molecular mechanisms implicating MCPyV-encoded proteins in cancer development are yet to be fully elucidated. This study builds upon our previous observations, which demonstrated that the MCPyV ST antigen enhances cell motility, providing a potential link between MCPyV protein expression and the highly metastatic nature of MCC. Here, we show that MCPyV ST remodels the actin cytoskeleton, promoting the formation of filopodia, which is essential for MCPyV ST-induced cell motility, and we also implicate the activity of specific Rho family GTPases, Cdc42 and RhoA, in these processes. Moreover, we describe a novel mechanism for the activation of Rho-GTPases and the cell motility pathway due to the interaction between MCPyV ST and the cellular phosphatase catalytic subunit PP4C, which leads to the specific dephosphorylation of β1 integrin. These findings may therefore provide novel strategies for therapeutic intervention for disseminated MCC.

## INTRODUCTION

Cell motility is a complex, multistep, and multicomponent process. The actin cytoskeleton is an important contributor to cell motility and is required for the formation of several types of actin-rich membrane protrusions (1). These protrusions contain actin filaments, which push the membrane forward, leading to membrane deformation and extension. Actin filaments are formed by the polymerization of globular monomeric actin (G-actin) into double-stranded helical filamentous actin (F-actin), which is enhanced by the activities of several actin-binding proteins (2). Actin filaments can adopt different morphologies, such as filopodia and lamellipodia, depending on the number of filaments and the type and number of actin-binding proteins that associate with these filaments (2). Filopodia are thin, finger-like structures that are filled with tight parallel bundles of F-actin. They have been described as “antennae” or “tentacles” that migrating cells use to probe their microenvironment, thus serving as pioneers during protrusion (3). In contrast, lamellipodia are thin, sheet-like protrusions filled with a branched network of actin. In both cases, the fast-growing barbed ends of actin filaments are oriented toward the plasma membrane, and the elongation of these protrusions pushes the leading edge forward, promoting cell migration (1).

This complex cell motility process occurs via the remodeling of the actin cytoskeleton, which is controlled by the Rho family GTPases, a large group of signaling molecules that act as signal mediators in the motility pathway. The Rho family GTPases affect both the microtubule network and the actin cytoskeleton (4). Three of the most widely studied Rho family GTPases, Cdc42, Rac1, and RhoA, have all been implicated in the formation of actin-containing plasma membrane protrusions. Cdc42 was originally implicated in filopodium formation (5), Rac1 in lamellipodium formation (6), and RhoA in stress fiber formation (7). While in many cases this classification is still valid, recent research has shown that the functions of these Rho family GTPases are intertwined, with cross talk in the cell motility cascade, for example, in filopodium formation (8). In addition to their important roles in healthy cells, Rho family GTPases also play roles in cancer development and metastasis (9–11). For instance, overexpression of RhoA has been observed in breast, colon, lung, and gastric cancers (12–14), among others.

Rho family GTPases are signal mediators and are central to the cell motility pathway. The pathway starts, however, with cellular receptors, such as the transmembrane integrin receptors (15). They are αβ heterodimers, with human integrins having 24 types of α subunits and 9 types of β subunits, although some types are found in specific tissues (e.g., α_3_β_2_ in platelets [16]) while others are widely expressed (e.g., α_5_β_1_) (17). Integrins have large extracellular domains that bind the extracellular matrix (ECM) and link to the actin cytoskeleton through short cytoplasmic tails (18). Their main function is to transmit signals from the ECM to the cell interior. In addition, integrins play a well-recognized role in cancer progression (19).

Cancers with high metastatic potential use the cell motility pathway to disseminate from the original tumor to distant secondary sites. One such cancer is Merkel cell carcinoma (MCC), a rare but aggressive malignancy of neuroendocrine origin that presents as reddish or purplish nodules on sun-exposed areas of skin (20). The number of reported cases of MCC has tripled in the past 20 years (21), and risk factors include advanced age, UV exposure, and immune suppression (20, 22). MCC has a poor 5-year survival rate, characterized by local recurrence, early spread to local lymph nodes, and high likelihood of forming distant metastases (20).

Merkel cell polyomavirus (MCPyV) is a recently discovered oncogenic virus that has been implicated as the causative agent of MCC in ∼80% of cases in the Northern Hemisphere (23). MCPyV infection is asymptomatic and ubiquitous in many populations, with up to 80% of healthy adults infected (24). Like other polyomaviruses, MCPyV expresses the T antigen, whose spliced products, namely, large T antigen (LT) and small T antigen (ST), are required for viral replication and tumorigenesis (23). The mechanism for MCPyV tumorigenesis has been broadly established (23, 25, 26). Upon loss of immunosurveillance due to old age or immunosuppression, the virus can integrate into the cellular genome. Integration occurs prior to clonal expansion of tumor cells (25). In addition to integration, another prerequisite for tumorigenesis is the truncation of MCPyV LT, as only the truncated form of the protein has been observed in MCPyV-positive MCC tumors. This is likely due to the fact that truncated MCPyV LT is replication deficient, as integrated MCPyV with a replication-competent MCPyV LT may initiate unlicensed replication, which would ultimately lead to cytopathic cell death (26). The molecular mechanisms of the MCPyV life cycle and oncogenic properties have been extensively reviewed (27–31).

Both LT and ST are required for MCPyV-positive MCC cell survival and proliferation, and small interfering RNA (siRNA)-mediated depletion of either leads to cell death (32). Taking simian virus 40 (SV40) as a model, MCPyV LT would be expected to be the main viral oncoprotein driving cellular transformation. However, in contrast to SV40, MCPyV LT cannot initiate cellular transformation (33), although it likely plays at least an accessory role, as it can bind to host factors that regulate cellular proliferation, such as the retinoblastoma protein (pRb) and Hsc70 (34). Conversely, MCPyV ST alone can transform rodent cells and induce serum-free proliferation of human cells, and it is therefore considered to be the main transforming factor (33). However, there have been conflicting results in regard to the contribution of MCPyV ST to MCPyV-positive MCC cell proliferation after initial transformation (35, 36), and thus, the role of MCPyV ST is not yet fully understood. What is known leads to the conclusion that MCPyV ST is a multifunctional protein. It promotes the hyperphosphorylation of 4E-BP1, deregulating cap-dependent translation (33); inhibits NF-κB-dependent gene transcription through NEMO (37); targets the host ubiquitin ligase SCF^Fwb7^, leading to the stabilization of MCPyV LT and several host oncoproteins (38); and also promotes transcriptional changes in glycolytic metabolic pathways (39). Importantly, recent studies using a panel of preterm transgenic mice coexpressing epidermis-targeted coexpression of MCPyV ST and the cell fate determinant atonal bHLH transcription factor 1 (ATOH1) led to the development of widespread cellular aggregates, with histology and marker expression mimicking human intraepidermal MCC, supporting the concept that ST is the major MCPyV-derived oncogenic driver in MCC (40).

MCC has a highly metastatic phenotype and correlates with poor MCC survival rates (41). This is also supported by recent studies showing that engraftment of MCC cell lines into SCID mice results in circulating tumor cells and metastasis formation (42). Aligned with this observation is our previous report that MCPyV ST can enhance cell motility through microtubule dissociation. Expression of MCPyV ST upregulates the levels of stathmin, a microtubule-associated protein, and leads to microtubule destabilization, which is necessary for a migratory phenotype (43).

Here, we extend this analysis and show that MCPyV ST drives cell motility by disrupting the actin cytoskeleton. We demonstrate that MCPyV ST expression induces the formation of filopodium-like structures through a mechanism dependent on the activities of the Rho family GTPases Cdc42, RhoA, and, to a lesser extent, Rac1. This process is initiated by an MCPyV ST-PP4C interaction that results in the dephosphorylation of β_1_ integrin.

## RESULTS

### MCPyV ST expression affects the levels of actin-associated proteins.

Cell motility regulation is a complex, multistep process, an important aspect of which is the regulation of the actin cytoskeleton and associated proteins. We have previously used a SILAC (stable isotope labeling by amino acids in cell culture)-based quantitative-proteomics approach (44, 45) to determine alterations in the host cell proteome upon inducible MCPyV ST expression in a HEK-293-derived cell line, i293-ST (43). The results demonstrated that MCPyV ST expression led to the upregulation of the microtubule-associated protein stathmin, which affects microtubule dissociation (46). Moreover, the results highlighted the fact that proteins that regulated the actin cytoskeleton were also altered upon MCPyV ST expression. These proteins include cofilin-1, cortactin, and actin-related protein 2/3 complex subunits, which were upregulated by 5.1-, 3.7-, and 3.9-fold, respectively.

**FIG 1:**
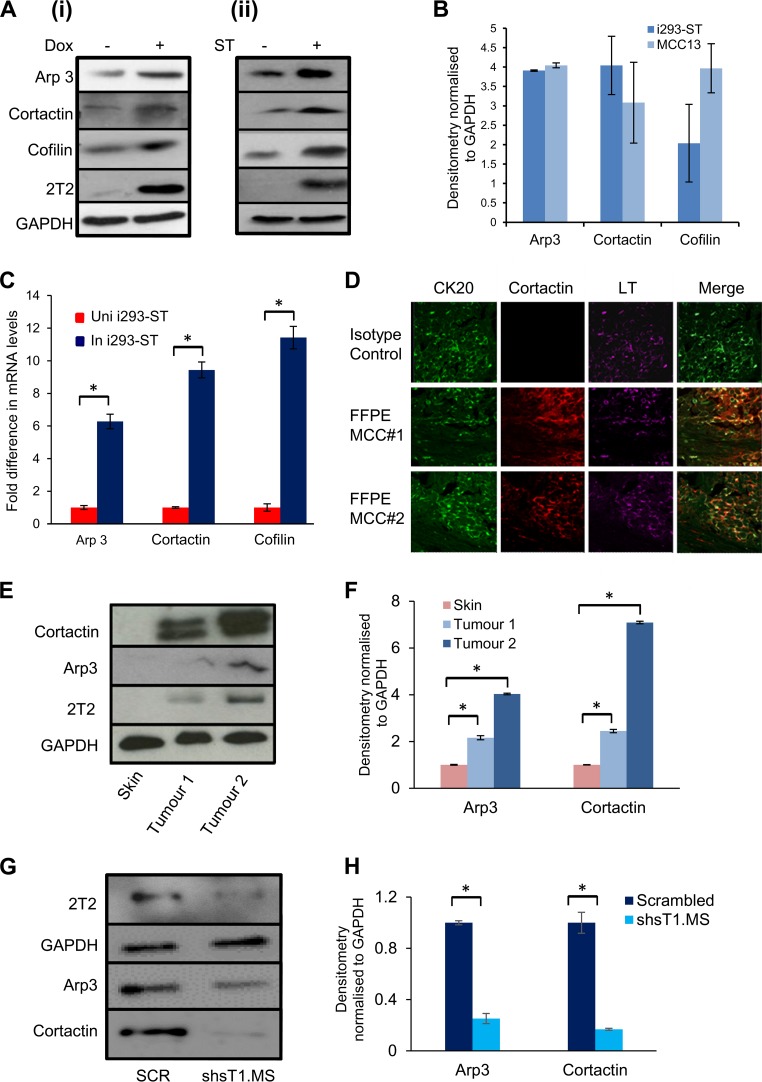
MCPyV ST expression results in the upregulation of several actin-associated proteins. (A) i293-ST cells remained uninduced or were incubated for 48 h in the presence of doxycycline hyclate (Dox) (i) or MCC13 cells were transfected with 1 μg EGFP or EGFP-ST for 12 h (ii). The cell lysates were then probed with Arp3-, cortactin-, and cofilin-specific antibodies. GAPDH was used as a measure of equal loading, and the 2T2 hybridoma was used to confirm MCPyV ST expression. (B) Densitometry quantification of the Western blots was carried out using Image J software and is shown as a percentage relative to the loading control, GAPDH (*n* = 3). (C) Total RNA was extracted from uninduced (Uni) or induced (In) i293-ST cells after 24 h, and relative transcript levels were analyzed by RT-qPCR using GAPDH as a reference. The fold increase was determined by ΔΔ*C_T_*, and statistical significance was analyzed using a nonpaired *t* test. Data from 3 independent experiments are presented as the fold increase versus uninduced control. *, *P* < 0.001. (D) FFPE sections of two primary MCC tumors were stained with CK20-, MCPyV LT-, and cortactin-specific antibodies or an isotype negative control. The sections were then incubated with Alexa Fluor-labeled secondary antibodies and analyzed using a Zeiss LSM 510 confocal laser scanning microscope. (E) Immunoblot analysis was performed on the cellular lysates of two independent MCC tumor samples and a negative-control nontumor cadaveric skin sample using Arp3- and cortactin-specific antibodies. GAPDH was used as a measure of equal loading, and the 2T2 hybridoma was used to confirm MCPyV ST expression. (F) Densitometry quantification of the Western blots was carried out using Image J software and is shown as a percentage relative to the loading control, GAPDH. The data were analyzed using three replicates per experiment (*n* = 3), and statistical analysis was done with a two-tailed *t* test with unequal variance. *, *P* < 0.01. (G) The MCPyV-positive MCC cell line WAGA was transduced with lentivirus expressing a scrambled shRNA or an ST-targeting shRNA. Upon ST depletion, the cell lysates were probed with Arp3- and cortactin-specific antibodies. GAPDH was used as a measure of equal loading, and the 2T2 hybridoma was used to confirm MCPyV ST expression. (H) Densitometry quantification of the Western blots was carried out using Image J software and is shown as a percentage relative to the loading control, GAPDH (*n* = 3). The error bars indicate standard deviations.

To confirm increased levels of actin-associated proteins identified by the quantitative-proteomics approach, i293-ST cells remained uninduced or were induced with doxycycline hyclate or an MCPyV-negative MCC cell line, MCC13 cells, were transfected with enhanced green fluorescent protein (EGFP) or an EGFP-tagged version of MCPyV ST (EGFP-ST). Immunoblotting of cell lysates confirmed upregulation of the actin-associated proteins Arp3, cortactin, and cofilin upon expression of MCPyV ST, in both i293-ST and MCC13 cells (Fig. 1A). Densitometry showed a 4-fold increase of Arp3 in both cell lines, an increase of cortactin by 4-fold in i293-ST cells and by 3-fold in MCC13 cells, and an increase of cofilin by 2-fold in i293-ST cells and by 4-fold in MCC13 cells (Fig. 1B). Comparing these values to those for control cells, a significant increase in actin-associated protein levels was observed in MCPyV ST-expressing cells. This increase in protein levels probably occurs at the transcriptional level, as reverse transcription-quantitative PCR (RT-qPCR) showed significant changes in the mRNA levels of Arp3, cortactin, and cofilin upon MCPyV ST expression in i293-ST cells (Fig. 1C), which correlates with recent results showing that MCPyV ST can dynamically alter the transcriptome of human cells (39).

To investigate the differential expression of actin-associated proteins in the context of MCC, multicolor immunochemistry analysis was performed on formalin-fixed, paraffin-embedded (FFPE) sections of two primary MCC tumors. The sections were incubated with cortactin-specific, cytokeratin 20 (CK20)-specific (a marker widely used to distinguish MCC), and MCPyV LT-specific (CM24B) antibodies. An isotype-matched control was also used as a negative control. The results showed increased levels of cortactin expression coincident with CK20 and LT staining in regions of both tumors (Fig. 1D). Moreover, immunoblot analysis was performed on the cellular lysates of two independent MCC tumor samples comparing protein levels to those in a negative-control nontumor cadaveric skin sample. The results again demonstrated an increase in cortactin and Arp3 protein levels in MCC tumor samples compared to the control (Fig. 1E and F). Notably, higher levels of actin-associated proteins were observed in MCC tumor sample 2 than in MCC tumor sample 1, which correlates with higher levels of MCPyV ST in sample 2. Furthermore, immunoblot analysis was also performed on cellular lysates of the MCPyV-positive MCC cell line WAGA, which were transduced with lentiviruses containing short hairpin RNA (shRNA) targeting ST or a scrambled control, as previously described (33, 38). The results demonstrated that depletion of MCPyV ST led to a reduction in cortactin and Arp3 protein levels (Fig. 1G). Together, these results demonstrate that levels of actin cytoskeleton-related proteins are altered upon MCPyV ST expression and in the context of MCC, implicating MCPyV ST in inducing cell motility and potentially MCC metastasis.

### MCPyV ST expression induces the formation of actin-based protrusions.

**FIG 2:**
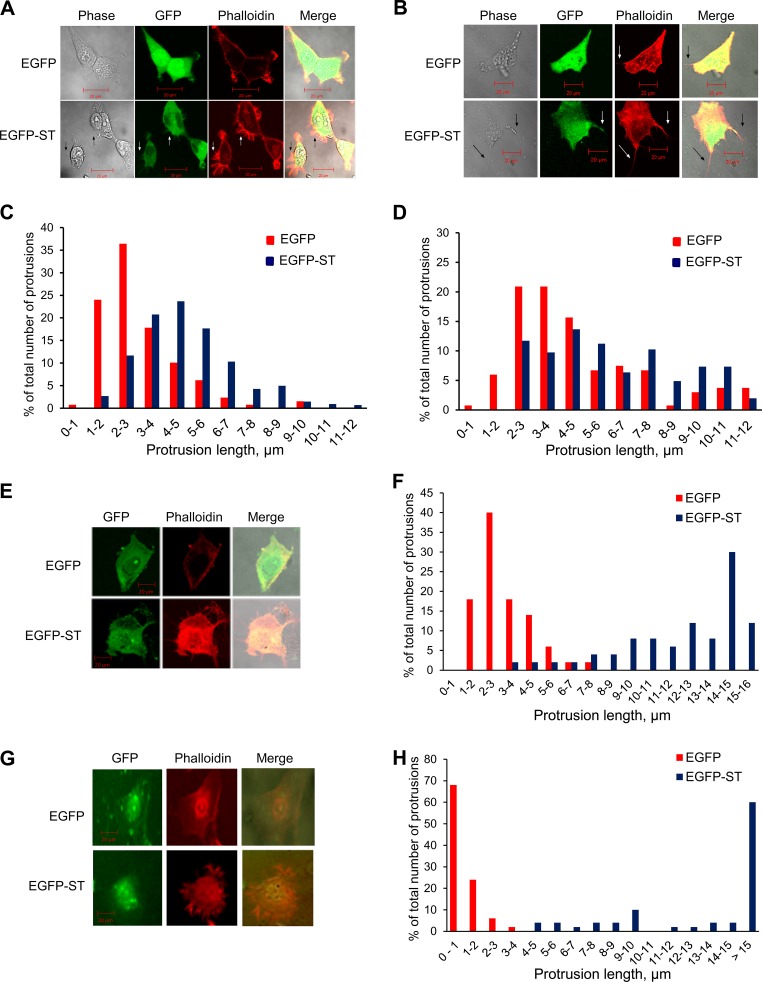
MCPyV ST expression results in an increase in the numbers and length of actin-based protrusions. (A, B, E, and G) HEK-293 (A) and MCC13 (B) cells, primary epidermal keratinocytes (E), and primary dermal fibroblasts (G) were transfected with 1 to 5 μg of either EGFP or EGFP-ST. The cells were fixed after 24 h and stained with rhodamine-phalloidin. The slides were then analyzed using a Zeiss LSM 700 confocal laser scanning microscope. (C, D, F, and H) The number and length of actin-based protrusions in each cell line were analyzed for 100 cells per condition using ImageJ software.

Observations of altered actin-associated protein levels upon MCPyV ST expression suggested the possibility of actin cytoskeleton-related phenotypic changes. To examine any changes in MCPyV ST-expressing cells, HEK-293 cells were transfected with EGFP or EGFP-ST. The cells were then fixed and stained with rhodamine-phalloidin, an actin-binding compound, to investigate any possible changes in the actin cytoskeleton. MCPyV ST-expressing HEK-293 cells showed an abundance of actin-based protrusions compared to the much smoother cell peripheries of control EGFP-expressing HEK-293 cells (Fig. 2A). Similar results were also observed in MCPyV ST-expressing MCC13 cells, showing an increased number of longer protrusions, although the difference in number was less pronounced. In addition, it was observed that MCC13 cells appeared to have more abundant intracellular actin, which may have been due to the increase in actin-associated protein levels upon MCPyV ST expression (Fig. 2B). To quantify the increase in actin-based protrusions upon MCPyV ST expression, the protrusions were counted and their lengths were measured using ImageJ software. Analysis showed an increase in the numbers and lengths of these actin-based protrusions in MCPyV ST-expressing HEK-293 cells (Fig. 2C) and an increase in longer actin-based protrusions in MCPyV ST-expressing MCC13 cells (Fig. 2D). Comparing the average lengths of protrusions from this analysis, a significant increase in length was observed in MCPyV ST-expressing cells. To confirm the actin cytoskeleton-related phenotypic changes observed in HEK-293 and MCC13 cells, similar experiments were performed in primary epidermal keratinocytes (Fig. 2E and F) and primary human dermal fibroblasts (Fig. 2G and H). The results in both primary cell lines showed an increase in the number and length of actin-based protrusions upon MCPyV ST expression. In addition, immunofluorescence studies suggested an increase in intracellular actin levels upon McPyV ST expression. Together, these observations suggest that MCPyV ST expression leads to phenotypic changes in the actin cytoskeleton, resulting in the formation of actin-based protrusions.

### MCPyV ST expression induces the formation of filopodia.

Visually, the thin, filamentous nature of the actin-based protrusions formed upon MCPyV ST expression suggests that they are filopodia. However, to conclusively characterize and classify these protrusions, a number of actin-associated proteins were screened using immunofluorescence. HEK-293 cells were cotransfected with Flag-tagged MCPyV ST (ST-Flag) (37) in combination with one of the green fluorescent protein (GFP)-tagged constructs (cortactin, N-WASP, mDia2, or fascin) or EGFP-ST and one of the myc-tagged constructs (IRSp53 or IRSTK). The cells were then fixed and stained with rhodamine-phalloidin to visualize actin-based structures.

**FIG 3:**
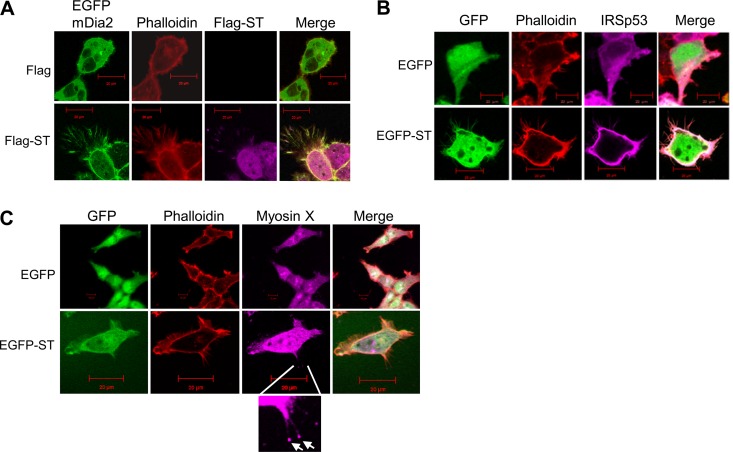
Screening of actin-associated proteins suggests MCPyV ST expression induces filopodium formation. (A) HEK-293 cells were cotransfected with 1 μg of EGFP-mDia2 and empty control vector or cotransfected with 1 μg of EGFP-mDia2 and ST-Flag. Twenty-four hours later, the cells were fixed and permeabilized, and GFP fluorescence was analyzed by direct visualization; in addition, the cells were stained with rhodamine-phalloidin and a Flag-specific antibody. (B) HEK-293 cells were cotransfected with 1 μg of EGFP and IRSp53-myc or cotransfected with 1 μg of EGFP-ST and IRSp53-myc. Twenty-four hours later, the cells were fixed and permeabilized, and GFP fluorescence was analyzed by direct visualization; in addition, the cells were stained with rhodamine-phalloidin and a Myc-specific antibody. (C) HEK-293 cells were transfected with 1 μg of EGFP or EGFP-ST. Twenty-four hours later, the cells were fixed and permeabilized, and GFP fluorescence was analyzed by direct visualization; in addition, the cells were stained with rhodamine-phalloidin and a myosin X-specific antibody. The enlarged box shows myosin X staining at the tips of filopodia (arrows). All the slides were analyzed using a Zeiss LSM 700 confocal laser scanning microscope.

Of particular interest were the results utilizing mDia2. In control cells, mDia2 was seen to be diffuse in the cytoplasm, while in cells expressing MCPyV ST, mDia2 relocalized to the cell periphery and into the actin-based protrusions (Fig. 3A). Similar results were observed for IRSp53, with the protein being observed to be diffuse in the cytoplasm in control cells but relocalizing to the cellular periphery and into the actin-based protrusions upon expression of MCPyV ST (Fig. 3B). No change in N-WASP or IRSTK could be observed upon MCPyV ST expression (data not shown). As both mDia2 (47) and IRSp53 (48) are associated with filopodium formation, this was the first marker-associated indication that MCPyV ST expression induced filopodium formation. To further confirm that MCPyV ST induced the formation of filopodia, an additional filopodium marker, myosin X, was utilized, as myosin X is localized to the tips of filopodia (49). To this end, HEK-293 cells were transfected with EGFP or EGFP-ST and stained for myosin X. Punctate foci of myosin X staining could be observed at the tips of MCPyV ST-induced actin-based protrusions (Fig. 3C). Together, these results confirmed that the protrusions induced by MCPyV ST expression were filopodia.

### The interaction between MCPyV ST and PP4C is important for inducing filopodium formation.

**FIG 4:**
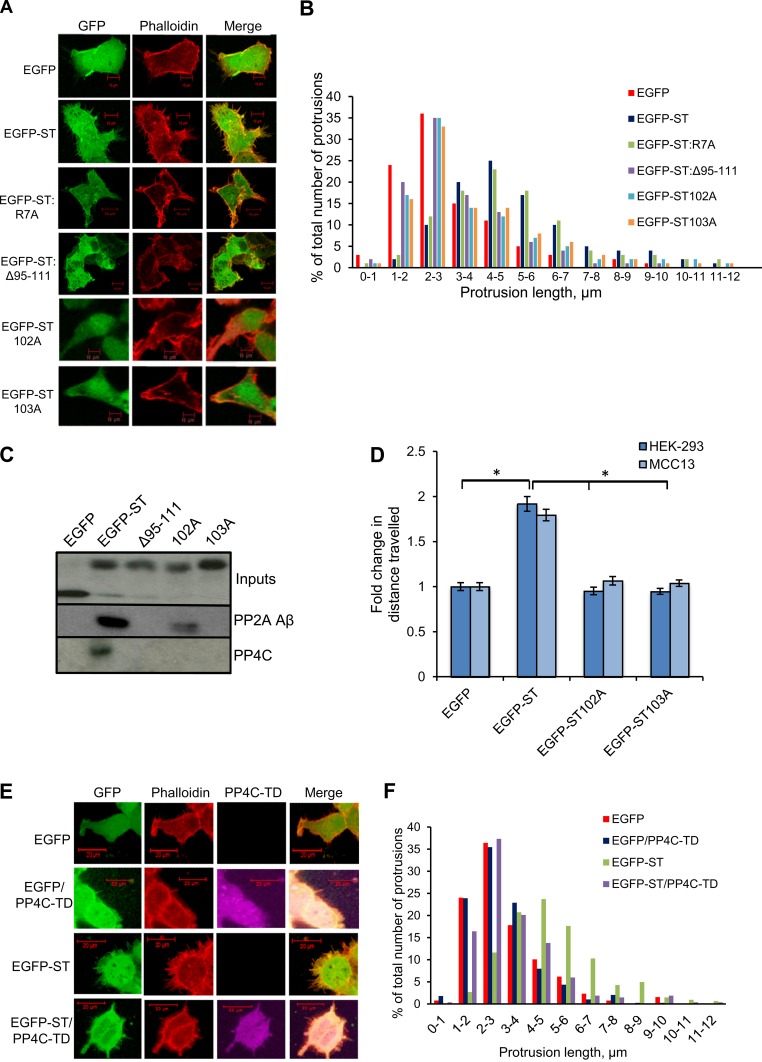
MCPyV ST interaction with cellular phosphatases is required for filopodium formation. (A) HEK-293 cells were transfected with 1 μg EGFP, EGFP-ST, EGFP-ST R7A, EGFP-STΔ95–111, EGFP-ST102A, or EGFP-ST103A. Twenty-four hours later, the cells were fixed, and GFP fluorescence was analyzed by direct visualization; in addition, the cells were stained with rhodamine-phalloidin. All the slides were analyzed using a Zeiss LSM 700 confocal laser scanning microscope. (B) The number and length of actin-based protrusions were analyzed for 50 cells per condition using ImageJ software. (C) HEK-293 cells were transfected with 1 μg of EGFP, EGFP-ST, EGFP-STΔ95–111, EGFP-ST102A, or EGFP-ST103A. Twenty-four hours later, the cell lysates were incubated with GFP-TRAP agarose beads. The pulldowns were then immunoblotted with PP2A Aβ- and PP4C-specific antibodies. A GFP-specific antibody was used to confirm the expression of the EGFP-tagged MCPyV ST constructs. (D) HEK-293 cells or MCC13 cells were transfected with 1 μg EGFP, EGFP-ST, EGFP-ST102A, or EGFP-ST103A. After 24 h, cell motility was analyzed using an IncuCyte kinetic live-cell-imaging system. Images were taken every 30 min over a 24-h period. Movement is represented as the average distance traveled compared to control EGFP-transfected cells (*n* = 25 per condition), and significance was tested using a 3-tailed Student *t* test. *, *P* < 0.01. The error bars indicate standard deviations. (E) HEK-293 cells were transfected with 1 μg of EGFP or EGFP-ST in the absence or presence of the PP4C transdominant (TD) mutant, PP4-RL. Twenty-four hours later, the cells were permeabilized and fixed, and GFP fluorescence was analyzed by direct visualization; in addition, the cells were stained with rhodamine-phalloidin and an HA tag-specific antibody. All the slides were analyzed using a Zeiss LSM 700 confocal laser scanning microscope. (F) The number and length of actin-based protrusions were analyzed for 50 cells per condition using ImageJ software.

We have previously shown the importance of the interaction between MCPyV ST and PP4C with regard to MCPyV ST-induced cell motility using a deletion mutant of MCPyV termed EGFP-STΔ95–111. This MCPyV ST mutant ablates the interaction between MCPyV ST and protein phosphatase 2A (PP2A) Aβ and PP4C (43). We therefore investigated whether cellular phosphatases were required for MCPyV ST-induced filopodium formation. For this purpose, HEK-293 cells were first transfected with either EGFP, EGFP-ST, EGFP-R7A (a previously described PP2A Aα nonbinding mutant), or EGFP-STΔ95–111 (PP2A Aβ and a PP4C nonbinding mutant). The cells were then fixed and stained with rhodamine-phalloidin to identify actin-based structures (Fig. 4A). The results suggested reduced filopodium formation upon the expression of EGFP-STΔ95–111 similar to that with the EGFP control, whereas EGFP-R7A induced filopodia similarly to EGFP-ST. Quantitative analysis of the filopodia confirmed that while expression of the EGFP-STΔ95–111 deletion mutant did not induce filopodia, expression of the MCPyV ST R7A mutant induced filopodium formation to levels similar to those with the wild-type ST (Fig. 4B). However, this analysis was not sufficient to determine which MCPyV ST-phosphatase interaction was important in cell motility and filopodium formation, as the Δ95–111 deletion affects the interaction of MCPyV ST with both PP2A Aβ and PP4C.

Therefore, to conclusively determine which cellular phosphatase is responsible for MCPyV ST-induced cell motility and filopodium formation, additional alanine-scanning MCPyV ST mutants were utilized to distinguish the interactions between MCPyV ST, PP2A Aβ, and PP4C, as previously characterized (50). However, these experiments utilized coimmunoprecipitation assays overexpressing tagged versions of PP2A and PP4C. Therefore, here, we repeated the coimmunoprecipitation experiments to examine the interaction of MCPyV ST-GFP mutants with endogenous PP2A Aβ and PP4C, using GFP-TRAP pulldown. The results show that wild-type EGFP-ST interacts with both endogenous forms of PP2A Aβ and PP4C. In contrast, EGFP-STΔ95–111 and EGFP-ST103A ablate both PP2A Aβ and PP4C binding, whereas EGFP-ST102A disrupts only the interaction with PP4C (Fig. 4C). Therefore, to determine which cellular phosphatase is responsible for MCPyV ST-induced cell motility, HEK-293 or MCC13 cells were transfected with either EGFP-ST102A or EGFP-ST103A, and the cells were imaged using the IncuCyte live-cell-imaging system (Fig. 4D). Cell motility was analyzed using ImageJ software by tracing the tracks of individual cells, allowing quantification of the distance traveled, and the results showed that in both cell lines expressing either EGFP-ST102A or EGFP-ST103A, a decrease in motility was observed in comparison to cells expressing EGFP-ST (*P* < 0.001) (Fig. 4D). This suggests that the specific interaction of MCPyV ST with PP4C is required for MCPyV ST-induced cell motility. To confirm the association between MCPyV ST, PP4C, cell motility, and filopodium formation, HEK-293 cells were transfected with EGFP-ST102A or EGFP-ST103A, fixed, and stained with rhodamine-phalloidin to observe actin-based structures (Fig. 4A). Together with quantitative analysis of filopodium formation (Fig. 4B), the results showed that cells expressing EGFP-ST102A and EGFP-ST103A had a marked decrease in filopodium formation compared to cells expressing EGFP-ST. Comparing the average lengths of protrusions from this analysis, a significant increase in length was observed in MCPyV ST- and R7A-expressing cells compared to EGFP-STΔ95–111-, EGFP-ST102A-, and EGFP-ST103-expressing cells. Together, this suggests that PP4C is required for MCPyV ST-induced filopodium formation. To confirm these observations, filopodium formation was also analyzed in the absence or presence of a PP4C transdominant phosphatase-dead mutant, PP4-RL (51). Analysis and quantification of rhodamine-phalloidin-stained cells showed a reduced number of longer actin-based protrusions in MCPyV ST-expressing cells in the presence of PP4-RL (Fig. 4E and F). Again, comparing the average lengths of protrusions from this analysis, a significant increase in length was observed in MCPyV ST-expressing cells alone compared to cells expressing PP4-RL. These results correlate with the data gathered from live-cell-imaging experiments and reveal the importance of the specific interaction of MCPyV ST with PP4C in inducing cell motility and filopodium formation.

### MCPyV ST-induced cell motility is dependent on the actions of Rho family GTPases.

**FIG 5:**
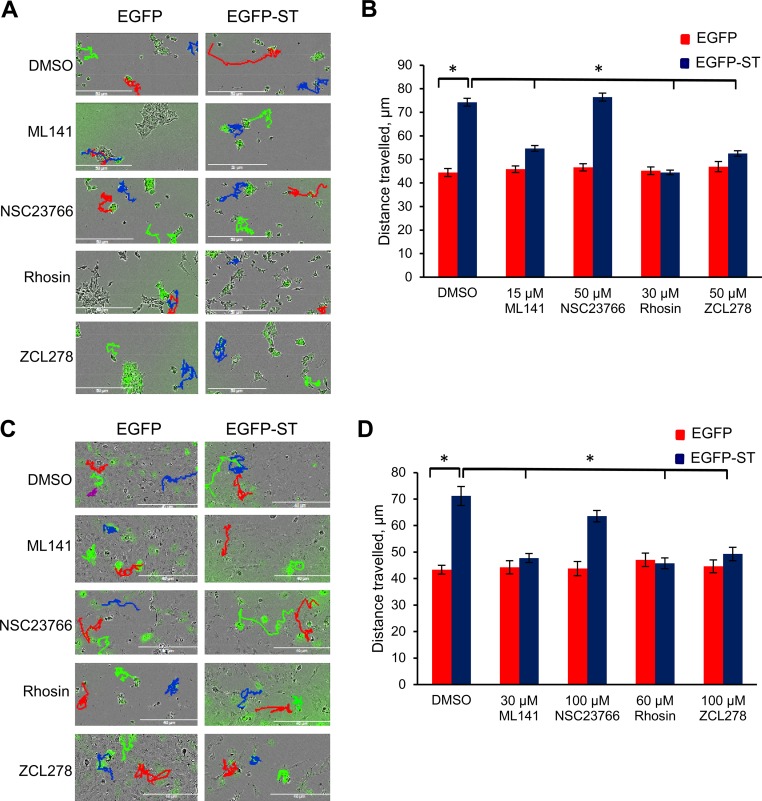
Live-cell images showed dependence of MCPyV ST-induced cell motility on Cdc42 and RhoA. (A and C) i293-EGFP and i293-EGFP-ST cells were induced using Dox (A), or MCC13 cells were transfected with 1 μg of EGFP and EGFP-ST (C). The cells were then treated with 1 μg/μl dimethyl sulfoxide (DMSO), 15 μM ML141, 50 μM NSC23766 or ZCL278, or 30 μM Rhosin. After 24 h, cell motility was analyzed using an IncuCyte kinetic live-cell-imaging system. Images were taken every 30 min over a 24-h period. The movements of the cells were then tracked using ImageJ software. (B and D) The average distance traveled was measured (*n* = 25 per condition), and significance was tested using a 3-tailed Student *t* test. *, *P* < 0.01. The error bars indicate standard deviations.

The Rho family GTPases are a superfamily of signaling molecules, some of which have been implicated in increased cell motility and metastasis in various cancers (9–11). There is also an undisputed role of the Rho family GTPases in actin dynamics (52). Therefore, to determine whether Rho family GTPases are involved in MCPyV ST-induced cell motility, a selection of well-characterized inhibitors targeting Rho family GTPases were utilized at noncytotoxic concentrations as measured by MTS [3-(4,5-dimethylthiazol-2-yl)-5-(3-carboxymethoxyphenyl)-2-(4-sulfophenyl)-2H-tetrazolium] assay (data not shown): ML141 (a Cdc42/Rac1 inhibitor), NSC23766 (a Rac1 inhibitor), Rhosin (a RhoA inhibitor), and ZCL278 (a Cdc42 inhibitor). Treatment of HEK-293-derived inducible cell lines, i293-EGFP and i239-EGFP-ST, alongside MCC13 cells enabled visualization of cell motility in live cells. i293-EGFP and i293-EGFP-ST cells were induced using doxycycline hyclate, while MCC13 cells were transfected with EGFP or EGFP-ST, and treated with each inhibitor for 24 h. The cells were then imaged using the IncuCyte live-cell-imaging system, taking an image every 30 min over a 24-h period (Fig. 5A and C). The distances traveled by individual cells were tracked using ImageJ software. No significant differences were observed among the average distances traveled by control cells not expressing MCPyV ST for both untreated cells and cells treated with the Rho family GTPase inhibitors (Fig. 5B and D), demonstrating that the concentrations of inhibitors were nontoxic. In contrast, decreased cell motility upon treating MCPyV ST-expressing cells with both Cdc42 inhibitors and the RhoA inhibitor was clearly apparent (*P* < 0.001), while no significant decrease was observed when cells were treated with the Rac1 inhibitor (Fig. 5B and D). This suggests a role for Cdc42 and RhoA GTPases in facilitating MCPyV ST-induced cell motility.

### MCPyV ST-induced filopodium formation is dependent on the activities of the Rho family GTPases.

**FIG 6:**
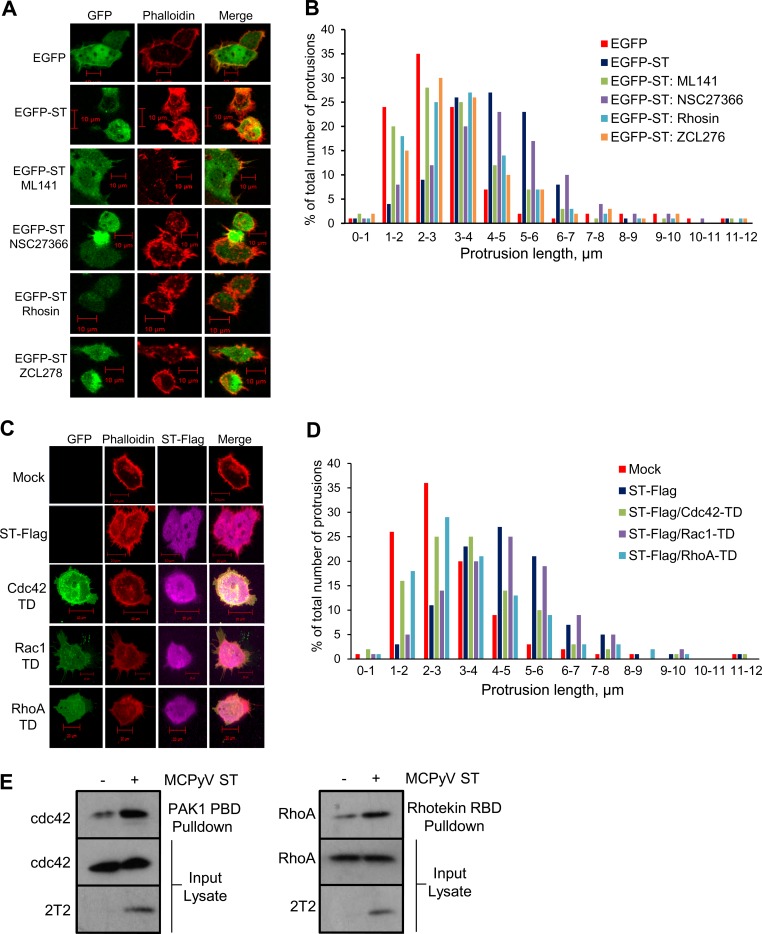
MCPyV ST-induced filopodium formation is dependent on the activities of Rho family GTPases. (A) HEK-293 cells were transfected with 1 μg EGFP or EGFP-ST and treated with 1 μg/μl DMSO, 15 μM ML141, 50 μM NSC23766 or ZCL278, or 30 μM Rhosin for 24 h. Then, the cells were fixed and GFP fluorescence was analyzed by direct visualization; in addition, the cells were stained with rhodamine-phalloidin. (C) HEK-293 cells were transfected with 1 μg EGFP-ST or cotransfected with 1 μg ST-Flag and pcDNA5-GFP-Cdc42-T17N, pcDNA5-GFP-Rac1-T17N, or pcDNA5-GFP-RhoA-T19N. Twelve hours later, the cells were fixed, permeabilized, and stained with rhodamine-phalloidin and a Flag-specific antibody. All the slides were analyzed using a Zeiss LSM 700 confocal laser scanning microscope. (B and D) The number and length of actin-based protrusions were analyzed for 50 cells per condition using ImageJ software. (E) HEK-293 cells were transfected with 1 μg EGFP or EGFP-ST, and after 24 h, the cell lysates were incubated with either PAK1 PBD or Rhotekin RBD agarose beads. The pulldowns were then immunoblotted with Cdc42- and RhoA-specific antibodies, and the 2T2 hybridoma was used to confirm MCPyV ST expression.

It was also important to determine whether the observed effects of Rho family GTPases on MCPyV ST-induced cell motility were mirrored in filopodium formation. HEK-293 cells were transfected with EGFP or EGFP-ST cells and treated with the specific Rho family GTPase inhibitors (ML141, NSC27366, Rhosin, and ZCL278) for 24 h. The cells were then fixed and stained with rhodamine-phalloidin to observe actin-based structures (Fig. 6A). Quantitative analysis of filopodia showed a decrease in filopodium formation when MCPyV ST-expressing cells were treated with Rho-GTPase inhibitors for Cdc42 and RhoA; however, the Rac1 inhibitor showed little effect (Fig. 6B). Comparing the average lengths of protrusions from this analysis, a significant increase in length was observed in MCPyV ST-expressing cells compared to cells treated with the Cdc42 and RhoA inhibitors.

To further examine any differences in MCPyV ST-induced filopodium formation by Rho family GTPases, HEK-293 cells were cotransfected with ST-Flag and the Rho family GTPase transdominant mutant pcDNA-5-GFP-Cdc42-T17N, pcDNA5-GFP-Rac1-T17N, or pcDNA5-GFP-RhoA-T19N (Fig. 6C). These transdominant mutants are inactive and inhibit endogenous Cdc42, Rac1, and RhoA activity (53). The cells were then fixed and stained with rhodamine-phalloidin to visualize actin-based structures. Quantitative analysis of filopodia showed a small decrease in filopodia upon coexpression of MCPyV ST with the Rac1 transdominant mutant; however, a marked reduction was observed when cells were cotransfected with either the Cdc42 or RhoA transdominant mutant, confirming the observation from the live-cell-imaging data (Fig. 6D). Again, comparing the average lengths of protrusions from this analysis, a significant increase in length was observed in MCPyV ST-expressing cells compared to cells expressing the Cdc42 or RhoA transdominant mutant. This suggests definitive roles for Cdc42 and RhoA in MCPyV ST-induced filopodium formation. Consequently, we next aimed to directly measure the activities of Cdc42 and RhoA in MCPyV ST-expressing cells, employing an affinity precipitation assay to specifically measure the amount of RhoA-GTP or Cdc42-GTP forms (54, 55). Transfected EGFP or EGFP-ST HEK-293 cell lysates were incubated with either PAK1 PBD or Rhotekin RBD agarose beads, which selectively bind to the GTP-bound, but not GDP-bound, forms of Cdc42 and RhoA, respectively. The amount of active G protein was then detected by immunoblotting with Cdc42- and RhoA-specific antibodies. Expression of MCPyV ST resulted in elevated levels of active Cdc42 and RhoA compared with control cells expressing GFP alone (Fig. 6E). Together, these data further indicate that the Rho family GTPases are involved in MCPyV ST-induced cell motility.

### The activities of integrins are important for MCPyV ST-induced cell motility.

**FIG 7:**
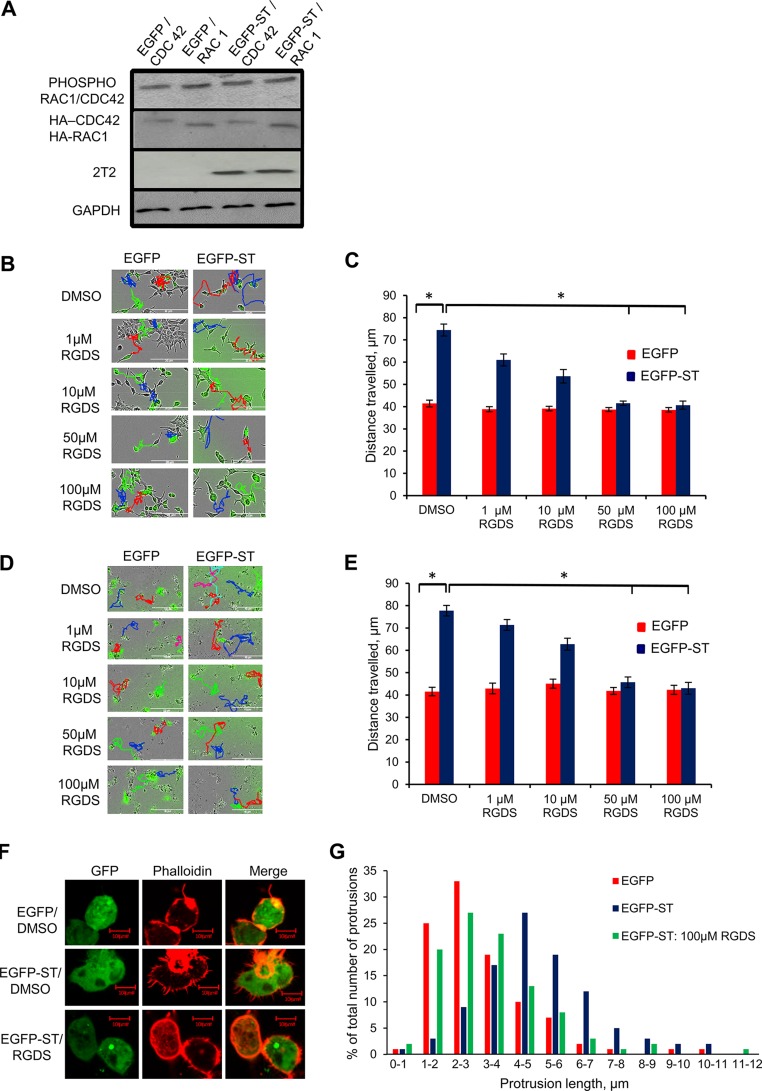
The integrin inhibitor RGDS reduces MCPyV ST-induced cell motility and filopodium formation. (A) i293-GFP or i293-GFP-ST cells were transfected with 1 μg HA-Cdc42 or HA-Rac1 and then induced with doxycycline hyclate for 6 h. The cell lysates were probed for phosphorylated Cdc42/Rac1 at the S71 residue. HA tag- and GAPDH-specific antibodies were used to measure equal loading. 2T2 was used to probe for MCPyV ST expression. (B and D) i293-EGFP and i293-EGFP-ST cells were induced using doxycycline hyclate (B), or MCC13 cells were transfected with 1 μg of EGFP and EGFP-ST (D). After 24 h, the cells were treated with 1 μg/μl DMSO or 1 μM, 10 μM, 50 μM, or 100 μM RGDS. After 24 h, cell motility was analyzed using an IncuCyte kinetic live-cell-imaging system. Images were taken every 30 min over a 24-h period. The movements of the cells were then tracked using ImageJ software. (C and E) The average distance traveled was measured (*n* = 25 per condition), and significance was tested using a 3-tailed Student *t* test. *, *P* < 0.001. (F) HEK-293 cells were transfected with 1 μg EGFP or EGFP-ST and then treated with 100 μM RGDS. After 24 h, the cells were fixed, and GFP fluorescence was analyzed by direct visualization; in addition, the cells were stained with rhodamine-phalloidin. (G) The number and length of actin-based protrusions were analyzed for 50 cells per condition using ImageJ software. The error bars indicate standard deviations.

Here and previously (43) we have reported the importance of the MCPyV ST-PP4C interaction in cell motility and filopodium formation. In order to uncover a potential role of PP4C in MCPyV ST-induced cell motility, i293-EGFP and i293-EGFP-ST cells were transfected with hemagglutinin (HA)-Cdc42 or HA-Rac1 and then induced for 48 h. The cell lysates were probed for phosphorylated Cdc42/Rac1 (residue Ser 71) to determine if MCPyV ST expression affects the phosphorylation status of Rho family GTPases. Surprisingly, the results showed no change in the phosphorylation status of Cdc42/Rac1 (Fig. 7A), indicating that the MCPyV ST-PP4C interaction does not affect the phosphorylation status of Rho family GTPases directly. Therefore, to uncover a possible target for the MCPyV ST-PP4C interaction, factors upstream of the Rho family GTPases were investigated. Integrins are known to be important in various aspects of cell adhesion, polarity, and motility, where they initiate signaling cascades (15), and have been implicated in cancer progression (19). Moreover, their function can be regulated by their phosphorylation status (56). Therefore, MCPyV ST-induced cell motility was assessed using a range of concentrations of the integrin inhibitor RGDS. RGDS is a tetrapeptide that has been shown to inhibit the binding of ligands to α_5_β_1_ and α_v_β_3_ integrins (57). i293-EGFP and i293-EGFP-ST cells were induced using doxycycline hyclate, while MCC13 cells were transfected with EGFP or EGFP-ST. The cells were then treated with nontoxic concentrations of RGDS as measured by a cell viability (MTS) assay (data not shown) for a 24-h period prior to imaging using the IncuCyte live-cell imaging system, taking an image every 30 min over a 24-h period (Fig. 7B and D). The distances traveled by individual cells were analyzed and showed decreasing cell motility with increasing RGDS concentration (Fig. 7C and E). These results therefore suggest a role for integrins in MCPyV ST-induced cell motility.

### The activities of integrins are important to MCPyV ST-induced filopodium formation.

To determine whether integrins have an essential role in MCPyV ST-induced filopodium formation, HEK-293 cells were transfected with EGFP or EGFP-ST and then treated with 100 μM RGDS for 24 h. The cells were then fixed and stained with rhodamine-phalloidin to visualize actin-based structures (Fig. 7F), coupled with quantitative analysis of filopodia (Fig. 7G). The results showed that cells expressing MCPyV ST and treated with RGDS displayed fewer filopodia than untreated cells expressing MCPyV ST alone. These results further imply a role for integrins in MCPyV ST-induced cell motility.

### The β_1_ integrin is dephosphorylated upon MCPyV ST expression.

**FIG 8:**
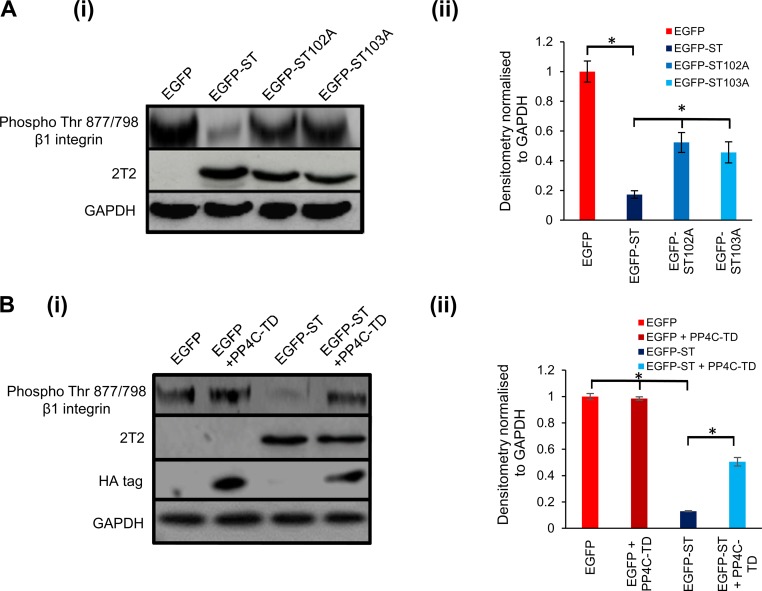
MCPyV ST expression reduces the phosphorylation levels of β_1_ integrin at Thr788/789 residues. (A) (i) HEK-293 cells were transfected with 1 μg EGFP, EGFP-ST, EGFP-ST102A, or EGFP-ST103A. After 24 h, the cell lysates were probed for phosphorylated Thr788/789 residues of β_1_ integrin. GAPDH was used to measure equal loading. 2T2 was used to probe for MCPyV ST expression. (ii) Densitometry quantification of the Western blots was carried out using Image J software and is shown as a percentage relative to the loading control, GAPDH. The data were analyzed using three replicates per experiment (*n* = 3), and statistical analysis was performed using a two-tailed *t* test with unequal variance. *, *P* < 0.01. (B) (i) HEK-293 cells were transfected with 1 μg EGFP or EGFP-ST in the absence or presence of the PP4C transdominant mutant, PP4-RL. After 24 h, the cell lysates were probed for phosphorylated Thr788/789 residues of β_1_ integrin. GAPDH was used to measure equal loading. 2T2 was used to probe for MCPyV ST expression. (ii) Densitometry quantification of the Western blots was carried out using Image J software and is shown as a percentage relative to the loading control, GAPDH. The data were analyzed using three replicates per experiment (*n* = 3), and statistical analysis was done using a two-tailed *t* test with unequal variance. *, *P* < 0.01. The error bars indicate standard deviations.

Integrin activity can be regulated by phosphorylation (56). The importance of integrins in MCPyV ST-induced cell motility and filopodium formation, as revealed above, suggested a possibility that integrin phosphorylation could be affected by MCPyV ST-PP4C interaction. In particular, the effects of RGDS on MCPyV ST-induced filopodium formation and cell motility suggested a role for α_5_β_1_ and/or α_v_β_3_. HEK-293 cells do not express α_v_β_3_ (58); thus, α_5_ and/or β_1_ were of interest. More specifically, the phosphorylation status of β_1_ has been shown to be regulated at the Thr788/789 residues (59). Therefore, in order to investigate whether MCPyV ST expression had any effect on β_1_ phosphorylation, HEK-293 cells were transfected with EGFP, EGFP-ST, EGFP-ST102A, or EGFP-ST103A. Cell lysates were probed for phosphorylated β_1_ integrin at Thr788/789. The results showed a dramatic reduction in the phosphorylation levels of β_1_ integrin at both sites upon MCPyV ST expression. Notably, however, phosphorylation levels remained unchanged upon the expression of the PP4C-nonbinding mutants EGFP-ST102A and EGFP-ST103A (Fig. 8). Densitometry results confirmed that upon MCPyV ST expression, the phosphorylation levels of β_1_ at Thr788/789 decreased (Fig. 8A). In contrast, this reduction was inhibited upon the expression of the PP4C transdominant mutant, PP4-RL (Fig. 8B). These results suggest that the interaction of MCPyV ST and PP4C leads to reduced β1 integrin phosphorylation at Thr788/789, which in turn leads to downstream signaling that ultimately enhances filopodium formation and cell motility in MCPyV ST-expressing cells.

## DISCUSSION

MCPyV ST is an oncogenic protein sufficient to transform rodent cells to anchorage- and contact-independent growth and is also capable of inducing serum-free proliferation of human cells (33). Moreover, epidermis-targeted coexpression of ST and ATOH1 leads to development of widespread cellular aggregates, with histology and marker expression mimicking those of human intraepidermal MCC, using a panel of preterm transgenic mice. This supports the concept that ST is the major MCPyV-derived oncogenic driver in MCC (40). Notably, MCC has a highly metastatic phenotype that correlates with poor MCC survival rates (41). We have confirmed the existence of a link between MCPyV ST expression and cell motility and migration, both essential factors for primary tumor dissemination. Our observations are supported by recent studies showing that engraftment of MCC cell lines into SCID mice resulted in the appearance of circulating tumor cells and metastasis formation, with explanted tumors also exhibiting an upregulation of MCPyV ST antigen expression in all tumors (42).

Promotion of motility and metastasis by virus oncoproteins has been reported previously. Human papillomavirus 16 (HPV16) E7, Epstein-Barr virus (EBV) EBNA1 and EBNA2, hepatitis B virus (HBV) X protein, and the SV40 ST have all been shown to induce metastasis through a variety of mechanisms, including disruption of cellular adhesion, cytoskeletal reorganization, and gene expression modulation (60–63). The utilization of the actin cytoskeleton in many viral processes, including cell transformation, has also been reported (reviewed in reference 64). In this report, we show that MCPyV ST also affects the actin cytoskeleton. Expression of MCPyV ST drives cell motility in a multistep process that involves the upregulation of a number of actin-associated proteins, forming filopodium-like structures through relocalization of filopodium-associated proteins.

The interaction of MCPyV ST with the Ser/Thr cellular phosphatases PP2A Aα, PP2A Aβ, and PP4C has been well documented (23, 37). Moreover, MCPyV ST interaction with PP4C seems to be important in promoting cell motility (43). PP4C has been implicated in apoptosis, DNA mutation, and cell proliferation (65), as well as a number of cell signaling pathways (66). In addition, it has been found to be upregulated in some cancers (67), and we have previously reported its involvement in destabilizing the microtubule network to promote cell motility (43). However, this is the first report of PP4C being implicated in actin dynamics, as MCPyV ST-induced filopodium formation is dependent upon the interaction.

We report that the Rho family GTPases appear to be involved in MCPyV ST-induced cell motility. Other oncogenic viruses have been reported to affect the Rho family GTPases. The best-known example is SV40 ST, whose activity leads to the rearrangement of filamentous actin networks, including Rac-induced lamellipodium formation, Cdc42 filopodium formation, and loss of RhoA-dependent stress fibers. Levels of Rac1 and Cdc42 are increased in cells expressing SV40 ST, while levels of RhoA are decreased (63). Thus, it is possible that a similar process occurs in cells expressing MCPyV ST, except through interaction with PP4C instead of PP2A. Our results also show that MCPyV ST motility seems to be dependent on Cdc42 and RhoA, as is MCPyV ST-induced filopodium formation. In addition, phosphorylation has been reported to negatively regulate the activity of RhoA (68, 69) and to affect the signaling of Cdc42 and Rac1 (70). However, our results suggest that MCPyV ST-PP4C interaction is not involved in modulating this effect directly.

Finally, we implicate integrins, including β_1_, in MCPyV ST-induced cell motility and filopodium formation. Integrins are cellular receptors that are known to be important in cell motility, particularly in Rho family GTPase cycling (71). Initially, an integrin inhibitor was used to observe whether integrins were important in MCPyV ST-specific cell motility. RGDS is a tetrapeptide found on fibronectin, fibrinogen α, and von Willebrand factor (72, 73), and it interacts with α_5_β_1_ and α_v_β_3_ integrins (57). Our results indicate that with increasing concentrations of RGDS, a reduction in MCPyV ST-induced cell motility is observed, and that RGDS also affects MCPyV ST-induced filopodium formation. We investigated β_1_ integrin, as it is expressed by HEK-293 cells. A number of studies have shown the significance of phosphorylation in the activities of integrins, including β_1_ (56, 59, 74). While the cellular phosphatase PP2A has been shown to dephosphorylate β_1_ integrin at Thr788/789 (59), our studies using the transdominant PP4C mutant and specific MCPyV ST mutants clearly showed a role for PP4C. Interestingly, in the absence of MCPyV ST, the PP4C transdominant mutant had no impact on integrin phosphorylation, indicating that PP4C function may be specifically redirected in the presence of MCPyV ST. Parallels can be found in the repurposing of key host factors by virus oncoproteins, for example, subversion of E6AP activity by the HPV E6 protein to ubiquitinate cellular p53. Our results show decreased Thr788/789 phosphorylation upon MCPyV ST expression and that this is dependent on the interaction of MCPyV ST with PP4C. We therefore suggest a mechanism where this interaction leads to the dephosphorylation of β_1_ integrin, which in turn activates the cell motility pathway. However, we cannot rule out the involvement of either other changes in the phosphorylation status of further integrins or, indeed, the other β_1_ integrin phosphorylation sites, for example, S785, which has been implicated in changes in cell motility in chicken cell lines (74).

**FIG 9:**
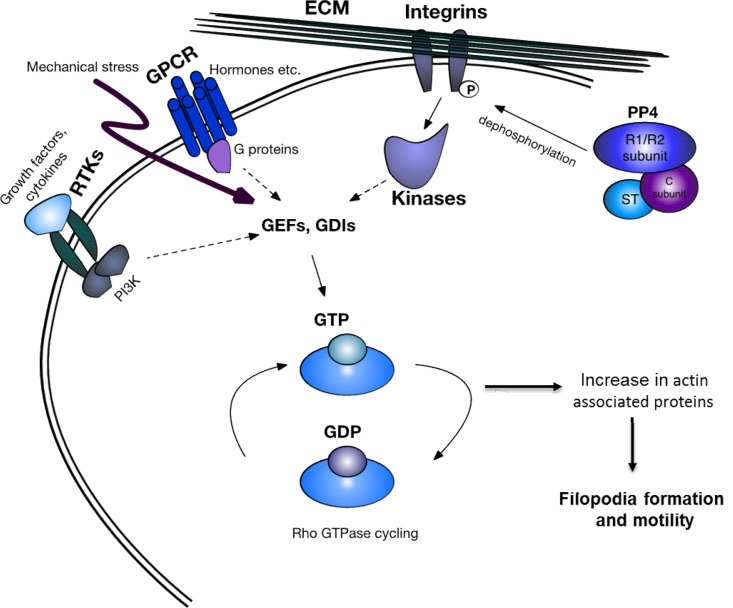
Schematic representation of MCPyV ST-induced cell motility. The MCPyV ST-PP4C interaction leads to the dephosphorylation of β_1_ integrin. This change in phosphorylation status leads to the initiation of the cell motility pathway, mediated via the Rho family GTPases. PI3K, phosphatidylinositol 3-kinase; ECM, extracellular matrix; GPCR, G-protein-coupled receptor; RTK, receptor tyrosine kinase.

Our overall findings suggest a possible mechanism (Fig. 9) whereby the interaction of MCPyV ST with PP4C leads to the dephosphorylation of one or more integrins, including β_1_. These changes may then contribute to the cell motility cascade through the Rho family GTPase modulators, leading to increased filopodium formation and cell motility. These findings highlight the importance of the MCPyV ST-PP4C interaction in promoting the metastatic phenotype of MCC. Therefore, this interaction may be a viable drug target. Currently, treatment of MCC depends on the disease stage, with surgical excision, lymph node dissection, and adjuvant radiotherapy as the standard. Metastasized MCC is treated with various regimens of broad-spectrum chemotherapy, such as anthracyclines, cyclophosphamide, etoposide, and platinum derivatives, alone or in combination. Over half of MCC patients respond to chemotherapy, but the median survival is 21.5 months (75). Potential virus-related drug targets are being identified, particularly for MCPyV LT and ST. Type I interferon (IFN) reduces LT expression and inhibits cell viability in MCPyV-positive MCC cell lines (76) but has failed to induce a clinical response in patients (77). In addition, YM155, an inhibitor of survivin, a cellular protein upregulated by MCPyV LT that is important for the survival of MCPyV-positive MCC cell lines, has shown a cytostatic effect in MCC xenograft tumors in mice (78, 79). Finally, the small-molecule tyrosine kinase inhibitor pazopanib (80) is currently undergoing phase II clinical trials. The field remains open for novel drugs.

In summary, we describe a novel mechanism by which a human tumor virus induces cell motility and cancer metastasis. As such, it provides new opportunities for therapeutic interventions for disseminated MCC.

## MATERIALS AND METHODS

### Plasmids and antibodies.

Expression vectors for EGFP-ST and EGFP-STΔ95–111 have been previously described (37, 43). EGFP-ST-R7A, EGFP-ST102A, and EGFP-ST103A were produced using the Q5 site-directed mutagenesis kit (New England BioLabs) according to the manufacturer's protocol and have been previously characterized (50). Sequence-verified mutants were cloned into EGFP using EcoRI and BamHI restriction sites. EGFP-mDia2 was provided by Shuh Narumiya, and pcDNA5-IRSp53-myc was provided by Laura Machesky. The transdominant mutants pcDNA3-GFP-Cdc42-T17N, pcDNA3-GFP-Rac1-T17N, and pcDNA3-GFP-RhoA-T19N were purchased from Addgene. MCPyV ST-tagging shRNA plasmids were kindly provided by Masa Shuda (Pittsburgh, PA). Antibodies for Arp3, cortactin, and cofilin were purchased from Genetex and used at 1:500 dilution; the p-Cdc42/Rac1 antibody (Cell Signaling Technologies), the p-β1-Thr788/789 antibody (Abcam), and myosin X (kindly provided by Michelle Peckham [University of Leeds]) were used at 1:100 dilution, and antibodies for Flag, myc, and HA (Sigma-Aldrich) and GFP (Living Colors) were used at 1:5,000 dilution. All the antibodies used for immunofluorescence were diluted 1:200.

### Chemicals.

Rho family GTPase inhibitors ML141 (Sigma-Aldrich), NSC23766 (Santa Cruz Biotech), ZCL278 (Tocris Bioscience), and Rhosin (Merck Milipore) were used at 15 μM, 50 μM, and 30 μM in i293-GFP and i293-GFP-ST and at 30 μM, 75 μM, and 60 μM in MCC13 cells. The integrin inhibitor RGDS (Tocris Bioscience) was used at a range of concentrations (see Results) on both 293-derived cells and MCC13 cells. Cell toxicity was measured using an MTS-based CellTiter 96 AqueousOne solution proliferation assay (Promega), as previously described (81).

### Mammalian cell culture.

The HEK-293 Flp-In cell line was purchased from Invitrogen. i293-ST (37), i293-GFP, and i293-GFP-ST cell lines were derived from HEK-293 Flp-In cells using the manufacturer's protocol, as previously described (37). HEK-293 cells (European Collection of Authenticated Cell Cultures [ECACC]) and derivative cells were grown in Dulbecco's modified Eagle's medium (DMEM) containing 10% fetal bovine serum (FBS) and 1% penicillin-streptomycin, as previously described (82). The MCPyV-positive MCC cell line WAGA, was grown in RPMI 1640 (Sigma) supplemented with 10% FBS. The MCC13 cell line (ECACC) was maintained in RPMI 1640 medium supplemented with 15% FBS and 1% penicillin-streptomycin. Primary normal human epidermal keratinocytes (Promocell) were cultured in serum-free keratinocyte medium (Gibco; 17005-34) supplemented with 5 ng/ml epidermal growth factor (EGF). Primary normal adult dermal fibroblasts (ATCC) were cultured using a fibroblast growth kit–serum-free kit (ATCC). ST-Flag, EGFP, and EGFP-ST expression was induced from i293-ST (34), i293-GFP, and i293-GFP-ST cells, respectively, with 2 μg/ml doxycycline hyclate for up to 48 h. The cells were plated in 6-well plates, and transfections routinely used 1 μg plasmid DNA and Lipofectamine 2000 (Life Technologies) or 5 μg plasmid DNA and nucleofection (Lonza), following the manufacturers' instructions.

### Multicolor immunohistochemistry.

FFPE sections from primary MCC tumors were purchased from Origene and analyzed as previously described (83). The primary antibodies were CK20 (Dako; dilution, 1:50), MCPyV LTA CM2B4 (Santa Cruz Biotechnology; dilution, 1:125), and anti-cortactin (Abcam; dilution, 1:250). An isotype-matched irrelevant antibody was used as a negative control on sections of tissues in parallel, and a rabbit polyclonal isotype control antibody (Abcam) was used to match the cortactin primary antibody. The sections were incubated with appropriate secondary antibodies labeled with different fluorochromes [Alexa Fluor 488 IgG2B and 633 IgG2A (Invitrogen) and IgG(H+L)-tetramethyl rhodamine isocyanate (TRITC) (Jackson ImmunoResearch)]. All slides were mounted with Immuno-Mount, and images were captured with a Zeiss LSM 510 confocal microscope.

### Immunoprecipitation assays and immunoblotting.

Coimmunoprecipitations, in addition to subsequent protein analysis by SDS-PAGE and Western blotting, were performed as previously described (84). Tumor and skin samples were homogenized in 5 volumes of suspension buffer {0.1 M NaCl, 10 mM Tris · Cl [pH 8.0], 1 mM EDTA, and 0.1 mg/ml AEBSF [4-(2-aminoethyl)benzenesulfonyl fluoride hydrochloride] protease inhibitor [Roche, Germany]}, as previously described (85). In contrast, cells were lysed in a modified radioimmunoprecipitation assay (RIPA) buffer (50 mM Tris-HCl, pH 7.6, 150 mM NaCl, 1% NP-40) supplemented with protease inhibitor cocktail (Roche) (73). For phosphorylation studies, cells were lysed in a modified buffer (20 mM Tris-HCl, pH 7.4, 150 mM NaCl, 50 mM NaF, 5 mM Na_4_O_7_P_2_B, 1 mM EDTA, 1 mM Ta_3_VO_4_, 10% glycerol, 1% Triton). Proteins were separated by SDS-PAGE before transfer onto nitrocellulose membranes (Hybond C extra; Amersham Biosciences). The membranes were probed with the appropriate primary and horseradish peroxidase (HRP)-conjugated secondary antibodies. Proteins were detected using EZ-ECL enhancer solution (Geneflow) as previously described (74). Densitometry was performed using ImageJ software.

### Live-cell imaging.

Cell motility was analyzed using an IncuCyte kinetic live-cell-imaging system as directed by the manufacturer. HEK-293 cells or i293-GFP/i293-GFP-ST cells were seeded at a density of 25,000 cells per well of a 6-well plate, and MCC13 cells were seeded at a density of 100,000 cells per well of a 6-well plate. After 12 h, the cells were transfected with 1 μg of DNA per well and/or induced using doxycycline hyclate. For transfected cells, the medium was changed after 6 h (HEK-293 or derivatives) or 12 h (MCC13). If appropriate, cells were treated with inhibitors for 24 h before imaging. Imaging was performed for a 24-h period, with images taken every 30 min. Cell motility was then tracked and analyzed using ImageJ software.

### Immunofluorescence.

Immunofluorescence assays were carried out as previously described (86). If appropriate, cells were treated with inhibitors for 24 h before fixing. The cells were viewed on a Zeiss LSM700 confocal microscope under an oil immersion 63× objective lens. Images were analyzed using LSM imaging software. Filopodia were counted using ImageJ software.

### Activation assay for RhoA and Cdc42.

The activation of RhoA or Cdc42 was determined with pulldown assays for activated RhoA or activated Cdc42, as previously described (87), using RhoA and Cdc42 activation assay kits (Cell Biolabs) as directed by the manufacturer's instructions. For the analysis of RhoA activation, cell lysates were incubated with Rhotekin RBD agarose beads, which have a high affinity for GTP-RhoA. For the analysis of Cdc42 activation, cell lysates were incubated with PAK1 PBD agarose beads, which have a high affinity for GTP-Cdc42. Affinity-precipitated activated GTP-bound RhoA or Cdc42 levels were then analyzed by immunoblotting using RhoA and Cdc42-specific antibodies.

### RT-qPCR.

RNA was extracted from uninduced and induced i293-ST cells using TRIzol (Invitrogen) (88). The RNA was DNase treated using the Ambion DNase-free kit according to the manufacturer's instructions, and RNA (1 μg) from each fraction was reverse transcribed with SuperScript II (Invitrogen) according to the manufacturer's instructions, using oligo(dT) primers (Promega). Ten nanograms of cDNA was used as the template in SensiMixPlus SYBR qPCRs (Quantace) according to the manufacturer's instructions, using a Rotor-Gene Q 5plex HRM platform (Qiagen) with a standard 3-step melting program (95°C for 15 s, 60°C for 30 s, and 72°C for 20 s), as previously described (89). With GAPDH (glyceraldehyde-3-phosphate dehydrogenase) as an internal control mRNA, quantitative analysis was performed using the comparative ΔΔ*C_T_* method, as previously described (90).
